# Connexin 37, 40, 43 and Pannexin 1 Expression in the Gastric Mucosa of Patients with Systemic Sclerosis

**DOI:** 10.3390/biomedicines11092487

**Published:** 2023-09-07

**Authors:** Berna Pavic, Marin Ogorevc, Katarina Boric, Dubravka Vukovic, Mirna Saraga-Babic, Snjezana Mardesic

**Affiliations:** 1 Renal Unit, University Hospital of Split, Šoltanska 1, 21000 Split, Croatia; berna.pavic@yahoo.com; 2 Department of Anatomy, Histology and Embryology, University of Split School of Medicine, Šoltanska 2, 21000 Split, Croatia; marin.ogorevc2@gmail.com (M.O.); msb@mefst.hr (M.S.-B.); 3 Department of Internal Medicine, University Hospital of Split, Šoltanska 1, 21000 Split, Croatia; kate.boric@gmail.com; 4 Department of Dermatovenerology, University Hospital of Split, Šoltanska 1, 21000 Split, Croatia; dglucina1@gmail.com

**Keywords:** connexin 37, connexin 40, connexin 43, pannexin 1, systemic sclerosis, gastric mucosa

## Abstract

Systemic sclerosis (SSc) is an autoimmune disease characterized by fibrosis of the skin and internal organs. Although its pathogenesis is not fully understood, connexins (Cxs) and pannexins (Panx) could be involved in the process of fibrosis. We analyzed the protein expression of Cx37, Cx40, Cx43, and Panx1 in the gastric mucosa of patients with SSc and healthy volunteers, using immunofluorescence staining. Protein levels of Cx37 were slightly increased, while the levels of Cx40 were significantly decreased in the lamina propria of the gastric mucosa of SSc patients compared to the controls. The changes were proportional to SSc severity, with the most prominent changes found in patients with severe diffuse cutaneous SSc. No differences in Cx43 or Panx1 levels were found between the analyzed groups of samples. The lack of changes in Cx43 expression, which has been previously associated with fibrosis, could be due to the weak expression of Cx43 in the gastric mucosa in general. Further studies on full-thickness gastric biopsies containing muscle layers and animal SSc models are needed to fully elucidate the role of Cxs and Panxs in SSc-associated fibrosis.

## 1. Introduction

Systemic sclerosis (SSc) is an autoimmune disease of connective tissue that is characterized by excessive deposition of extracellular matrix (ECM) into the skin and internal organs, with the other two main features being autoantibody production and vascular injury [1,2]. Although a definitive etiology of the disease remains undetermined, several risk factors are known, such as female gender, African-American race, advanced age, family history of SSc, and exposure to silica particles and various solvents [3,4,5,6]. Depending on the extent of cutaneous involvement, SSc is classified as limited cutaneous SSc (lcSSc) if there is no history of skin involvement proximal to the elbows and knees, or diffuse cutaneous SSc (dcSSc) if the opposite is true [2]. Individuals with dcSSc have a higher risk of developing internal organ involvement compared to those with lcSSc [7]. The most common internal organ complications of SSc are gastrointestinal manifestations [8]. While gastric manifestations stemming from vascular ectasia and dysmotility are variable [9], gastric involvement in general is associated with increased morbidity and mortality [10].

Although the exact pathogenesis of SSc has not been fully elucidated, fibrosis is considered to play a major role in its symptoms [11]. Fibrosis is a process that involves an inflammatory response and transient fibroblast activation, resulting in cell proliferation and production of high amounts of ECM [12]. Studies have shown that fibroblast activation is mediated by cytokines, such as transforming growth factor beta (TGF-β), secreted by infiltrating immune cells [13]. Fibroblasts isolated from SSc lesions differ from those isolated from healthy skin, showing increased expression of fibrosis-associated factors such as collagens, adhesion molecules and metalloproteinase inhibitors [14,15,16]. SSc fibroblasts have the characteristics of healthy fibroblasts activated by TGF-β [17], implying TGF-β as a main mediator of fibrosis in SSc, which is further supported by the fact that elevated levels of TGF-β have been described in patients with SSc [18].

Connexins (Cxs) are transmembrane proteins with four transmembrane domains, two extracellular loops, and cytoplasmic amino and carboxyl tails [19]. Six connexin proteins join together to form a hemichannel (connexon),while two hemichannels from neighboring cells dock with each other to form a gap junction [20]. Pannexins (Panxs) are also transmembrane proteins with tetraspan topography that form hemichannels, but do not form gap junctions [21]. While connexins mainly mediate direct intercellular communication via gap junctions, pannexins are primarily involved in the transfer of intracellular molecules to extracellular space [22].

Considering that changes in Cx and Panx expressions have been associated with fibrosis, in the present study we hypothesized that the expression of Cxs and Panx1 would differ between the gastric mucosa of healthy individuals and SSc patients, especially in those with severe dsSSc. The aim of this study was to investigate and compare the protein expression of Cx37, Cx40, Cx43, and Panx1 in the gastric mucosa samples taken from patients with different forms of SSc and healthy volunteers. Our findings could determine which, if any, of the analyzed proteins are altered in SSc, and such results could contribute to the further understanding of the role of Cx and Panx in SSc pathogenesis.

## 2. Materials and Methods

This study was approved by the Ethical and Drug Committee of the University Hospital of Split (class: 500-03/18-01/55; registry number: 2181-147-01/06/M.S.-18-2) and the Ethical Committee of the University of Split School of Medicine (class: 003-08/19-03/0003; registry number: 2181-198-03-04-19-0009), and the procedures follow all relevant guidelines of the Declaration of Helsinki [23].

### 2.1. Patients

A total of 20 female Caucasian patients were enrolled in the study after giving written informed consent. Fifteen patients had a diagnosis of SSc according to the criteria of the American College of Rheumatology/European League Against Rheumatism [24], while five healthy volunteers (controls, CTRL) were matched for age and sex (Table 1). Criteria for exclusion were the presence of other autoimmune diseases, infection, malignancy, treatment with intravenous immunoglobulins or immunosuppressive therapy, and the use of more than 10 mg per day of prednisone or an equivalent or any therapy affecting the gastric mucosa (proton pump inhibitors, histamine H2-receptor blockers, nonsteroidal anti-inflammatory drugs, etc.) within 6 months prior to tissue sampling. Depending on the extent of skin involvement assessed using the modified Rodnan skin score [25], the patients with SSc were divided into two groups as described by LeRoy et al. [2]: 5 patients had lcSSc (LC), and 10 patients had dcSSc. The patients with dcSSc were further divided on the basis of the severity of organ involvement assessed with Medsger’s severity score [26]: 5 patients had moderate dcSSc (SYS1), and 5 had severe dcSSc (SYS2).

### 2.2. Tissue Procurement and Processing

The tissue samples were gathered as previously described [27]. Biopsies of the gastric mucosa were collected from each patient using upper gastrointestinal endoscopy. Endoscopic mucosal resections of the corpus of the stomach were performed using pincers (Olympus Medical Systems Co Ltd., Tokyo, Japan), and the tissue was immediately submerged in 4% paraformaldehyde for fixation. Subsequently, the tissue was dehydrated using graded ethanol solutions, cleared using xylol, and embedded in paraffin. Using a microtome, 5 µm thick serial sections were made from the paraffin blocks and mounted on glass slides. Every tenth section was stained by hematoxylin and eosin to confirm the preservation of tissue morphology.

### 2.3. Double Immunofluorescence Staining

The immunofluorescence staining protocol was performed as described previously [28]. Briefly, the tissue slides were deparaffinized in xylol and rehydrated in graded ethanol solutions. The samples were then heated in sodium citrate buffer (pH 6.0) using a steam cooker for 30 min. After a round of washing in phosphate-buffered saline (PBS), protein blocking buffer (Protein Block ab64226, Abcam, Cambridge, UK) was applied for 20 min in a humid chamber. Combinations of primary antibodies (Table 2) were applied and incubated overnight in the humid chamber. The primary antibodies were rinsed by washing the samples in PBS, and appropriate secondary antibodies (Table 2) were applied for 1 h in the humid chamber. Afterward, samples were once again washed in PBS, counterstained with 4′,6-diamidino-2-phenylindole (DAPI) for 2 min, and cover-slipped using a mounting medium (ImmuMount, Thermo Shandon, Pittsburgh, PA, USA). The specificity of staining was controlled by omitting primary antibodies from the staining protocol. The stained samples were analyzed using an epifluorescence microscope (Olympus BX61, Tokyo, Japan), and images were captured with a mounted digital camera (Nikon Ri-D2, Nikon, Tokyo, Japan) using NIS-Elements F software version 3.0 (Nikon, Tokyo, Japan).

### 2.4. Immunofluorescence Signal Quantification

In order to quantify the immunofluorescence signal of the analyzed proteins, we calculated the area percentage that the signal took up in the captured images. For each sample, we captured two representative nonadjacent images with a 40× objective, one from the superficial region and the other from the deeper region of the mucosa. This gave a total of 10 images for analysis for each of the 4 sample groups (ctrl, lc, sys1, sys2). Each image was processed in the following way: First, using Adobe Photoshop version 21.0.2 (Adobe, San Jose, CA, USA) the background signal was removed using the “levels” function. Then, the epithelium was selected using the Lasso tool and separated from the lamina propria by cutting it from the original image and placing it into a blank image of the same dimensions. The separated images were then opened in ImageJ software version 1.53o (NIH, Bethesda, MD, USA), and the red color channel was subtracted to purify the green signal. Images were duplicated, and the median filter was applied to one of the images (with radius 5 for Cx37 and Cx43, and radius 10 for Cx40 and Panx1). The filtered images were subtracted from the nonfiltered images to isolate the positive signal. The resulting images were turned into 8-bit images and thresholded using the “triangle” method. The area percentage of the thresholded images was determined using the “analyze particles” function. Considering that parts of all analyzed images were devoid of any tissue, the measured area percentage was lower than the actual area percentage. To correct the area percentage value, we determined the number of total pixels (px) of the images and the number of empty space pixels using the Magic Wand tool in Adobe Photoshop. The corrected area percentage was calculated using the following formula:

$$\mathrm{Corrected}\;\mathrm{area}\;\mathrm{percentage}=\frac{\mathrm{Uncorrected}\;\mathrm{area}\;\mathrm{percentage}\;\times \;\mathrm{total}\;\mathrm{px}}{\mathrm{total}\;\mathrm{px}\;-\;\mathrm{empty}\;\mathrm{space}\;\mathrm{px}},$$

and it was used for the statistical analyses. To further characterize the protein expression in the lamina propria, we separated the cells into two categories: αSMA-positive and αSMA-negative. On the same images used for area percentage calculation, we determined the percentage of αSMA-positive lamina propria cells that also co-expressed the analyzed proteins by overlapping fluorescence images in Adobe Photoshop, and the same was applied for αSMA-negative cells.

### 2.5. Statistical Analysis

Statistical analysis was performed using GraphPad Prism version 9.0.0. software (GraphPad Software, San Diego, CA, USA). All results are presented as the mean and standard deviation of the calculated percentages. The normality of distribution of the data was determined using the Shapiro–Wilk test. Two-way analysis of variance (ANOVA) with Tukey’s post hoc test was used to determine the statistical significance of the difference in protein expression between the analyzed groups of samples. Statistical significance was set at *p* < 0.05.

## 3. Results

The healthy gastric mucosa of control samples (CTRL) consisted of the simple columnar surface epithelium and underlying lamina propria containing gastric glands. Invaginations of the surface epithelium (gastric pits) and the superficial parts of gastric glands displayed a typical columnar epithelial appearance (mainly mucus cells), while the deeper parts of gastric glands consisted of a cuboidal-like epithelium (parietal and chief cells). For the area percentage analysis, the surface epithelium, epithelial cells of gastric pits, and all parts of gastric glands were grouped as one category—epithelium. αSMA staining characterized the smooth muscle cells of blood vessels and myofibroblasts found in the lamina propria, while blue DAPI staining characterized all cell nuclei.

In lcSSc and dsSSc samples, histological changes included hyalinization of the lamina propria, defects of the surface epithelium, and lymphocyte accumulations (a more detailed description can be found in our previous study [27]).

### 3.1. Double Immunofluorescence Staining for Cx37 and αSMA

**Figure 1:**
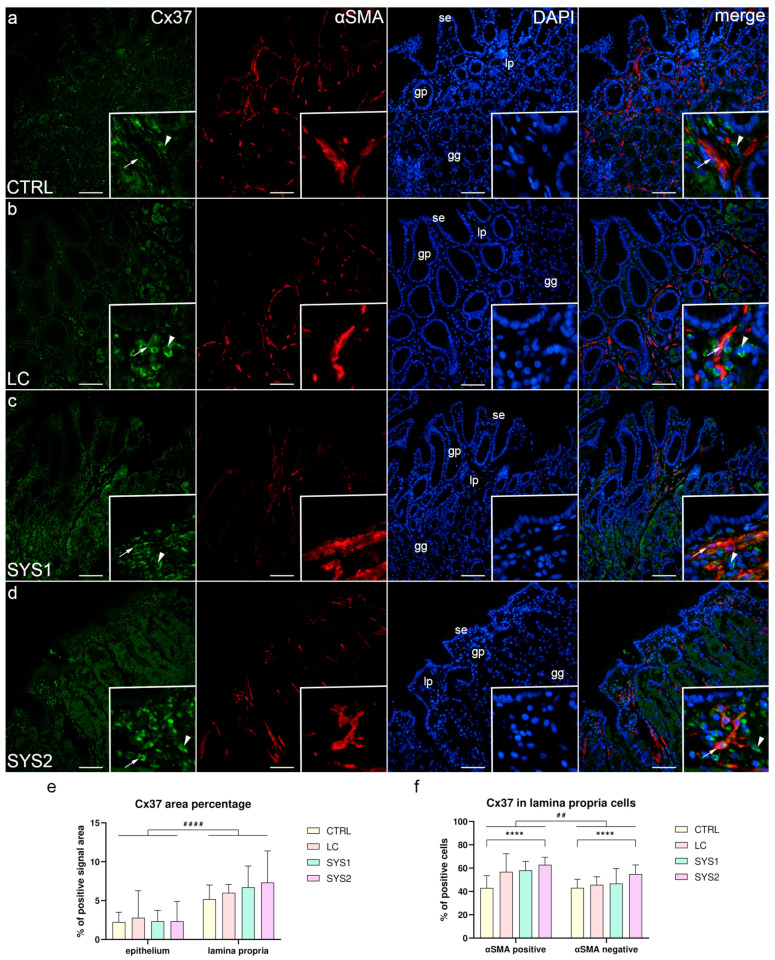
Co-expression of Cx37 and αSMA in the gastric mucosa of patients with SSc. CTRL—control samples from healthy volunteers (**a**); LC—samples from patients with limited cutaneous SSc (**b**); SYS1—samples from patients with moderate diffuse cutaneous SSc (**c**); SYS2—samples from patients with severe diffuse cutaneous SSc (**d**). se, surface epithelium; gp, gastric pits; gg, gastric glands; lp, lamina propria. Insets display Cx37 expression in lamina propria cells (**a**–**d**). Arrows mark αSMA-positive cells expressing Cx37, while arrowheads demonstrate αSMA-negative cells with Cx37 expression (**a**–**d**). DAPI staining shows all cell nuclei (**a**–**d**). Double immunofluorescence staining for Cx37 and αSMA, 200× magnification, scale bars: 200 µm. The graphs display differences in Cx37 area percentage of epithelium and lamina propria (**e**), as well as differences between αSMA-positive and -negative cells of the lamina propria (**f**). Error bars mark the standard deviation. Asterisks (*) symbolize significant differences between sample groups, while hashes (#) represent differences between analyzed regions/cells. ^##^ *p* < 0.01, ****/^####^ *p* < 0.0001.

The surface epithelium, gastric pits, and superficial parts of gastric glands of CTRL samples showed weak Cx37 staining, mainly in their basal membrane. However, some cells of the deeper parts of gastric glands displayed moderate Cx37 staining., while several cells in the lamina propria, both αSMA-positive and αSMA-negative, demonstrated strong Cx37 staining (Figure 1a). In LC samples, Cx37 had a similar expression pattern in the epithelium, gastric glands, and lamina propria to CTRL samples, with a slightly higher proportion of αSMA-positive cells also co-expressing Cx37 (Figure 1b). Cx37 expression in SYS1 samples was almost identical to LC samples, in both epithelial and lamina propria cells (Figure 1c). While the surface and glandular epithelium of SYS2 samples showed no differences in Cx37 staining compared to other sample groups, the lamina propria had the highest proportion of Cx37-positive cells (Figure 1d). While statistical analysis of the Cx37 area percentage of all samples showed no significant differences between sample groups, the lamina propria had a significantly higher (*p* < 0.0001) area percentage compared to the epithelium across all sample groups (Figure 1e). While the proportion of Cx37-positive lamina propria cells increased with the severity of SSc, only SYS2 samples showed a significantly higher proportion (*p* < 0.0001) compared to CTRL samples, for both αSMA-positive and -negative cells. αSMA-positive cells had significantly higher (*p* = 0.001) Cx37 positivity compared to αSMA-negative cells when analyzing across all sample groups (Figure 1f).

### 3.2. Double Immunofluorescence Staining for Cx40 and αSMA

**Figure 2:**
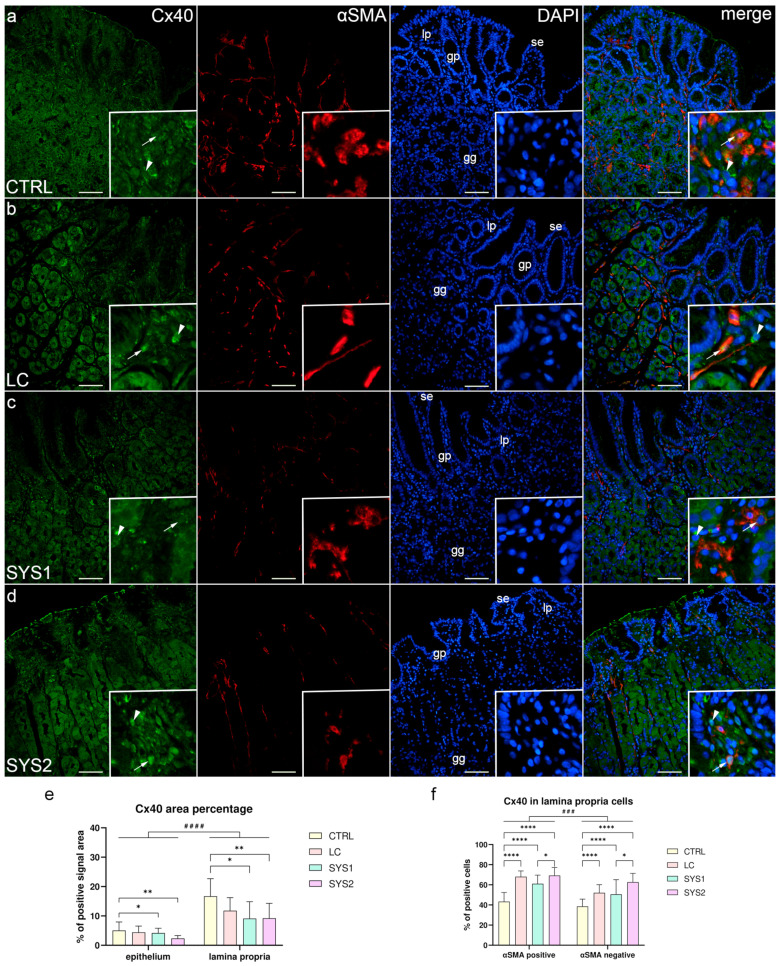
Co-expression of Cx40 and αSMA in the gastric mucosa of patients with SSc. CTRL—control samples from healthy volunteers (**a**); LC—samples from patients with limited cutaneous SSc (**b**); SYS1—samples from patients with moderate diffuse cutaneous SSc (**c**); SYS2—samples from patients with severe diffuse cutaneous SSc (**d**). se, surface epithelium; gp, gastric pits; gg, gastric glands; lp, lamina propria. Insets display Cx40 expression in lamina propria cells (**a**–**d**). Arrows mark αSMA-positive cells expressing Cx40, while arrowheads demonstrate αSMA-negative cells with Cx40 expression (**a**–**d**). DAPI staining shows all cell nuclei (**a**–**d**). Double immunofluorescence staining for Cx40 and αSMA, 200× magnification, scale bars: 200 µm. The graphs display differences in Cx40 area percentage of epithelium and lamina propria (**e**), as well as differences between αSMA-positive and -negative cells of the lamina propria (**f**). Error bars mark the standard deviation. Asterisks (*) symbolize significant differences between sample groups, while hashes (#) represent differences between analyzed regions/cells. * *p* < 0.05, ** *p* < 0.01, ^###^ *p* < 0.001, ****/^####^ *p* < 0.0001.

CTRL samples displayed strong Cx40 positivity in both the apical and the basal regions of (the basal membrane) some cells in the surface epithelium, gastric pits, and superficial parts of gastric glands. Some cells of the deeper parts of gastric glands showed strong (membranous) basal Cx40 staining, while other cells had weak or no staining at all. Strong Cx40 staining was also present in αSMA-positive and -negative cells of the lamina propria (Figure 2a). Cx40 staining of the epithelium and gastric glands in LC samples was identical to CTRL samples, while the lamina propria had comparatively more positive cells; however, their staining was weaker (Figure 2b). Both SYS1 and SYS2 samples had fewer Cx40-positive cells in their gastric glands, while they had more positive cells in the lamina propria compared to CTRL samples. The Cx40 staining of the lamina propria cells was weak in SYS1 samples (Figure 2c) and moderate in SYS2 samples (Figure 2d). Statistical analysis of the Cx40 area percentage showed a significantly lower area percentage in SYS1 (*p* = 0.0391) and SYS2 samples (*p* = 0.0056) compared to CTRL samples, for both the epithelium and the lamina propria. The lamina propria had a significantly higher (*p* < 0.0001) area percentage compared to the epithelium across all sample groups (Figure 2e). The proportion of Cx40-positive lamina propria cells was higher in all SSc patients compared to healthy volunteers (*p* < 0.0001), and SYS2 samples had a significantly higher proportion than SYS1 samples (*p* = 0.016), for both αSMA-positive and αSMA-negative cells. αSMA-positive cells had significantly higher (*p* = 0.0004) Cx40 positivity compared to αSMA-negative cells when analyzing across all sample groups (Figure 2f).

### 3.3. Double Immunofluorescence Staining for Cx43 and αSMA

**Figure 3:**
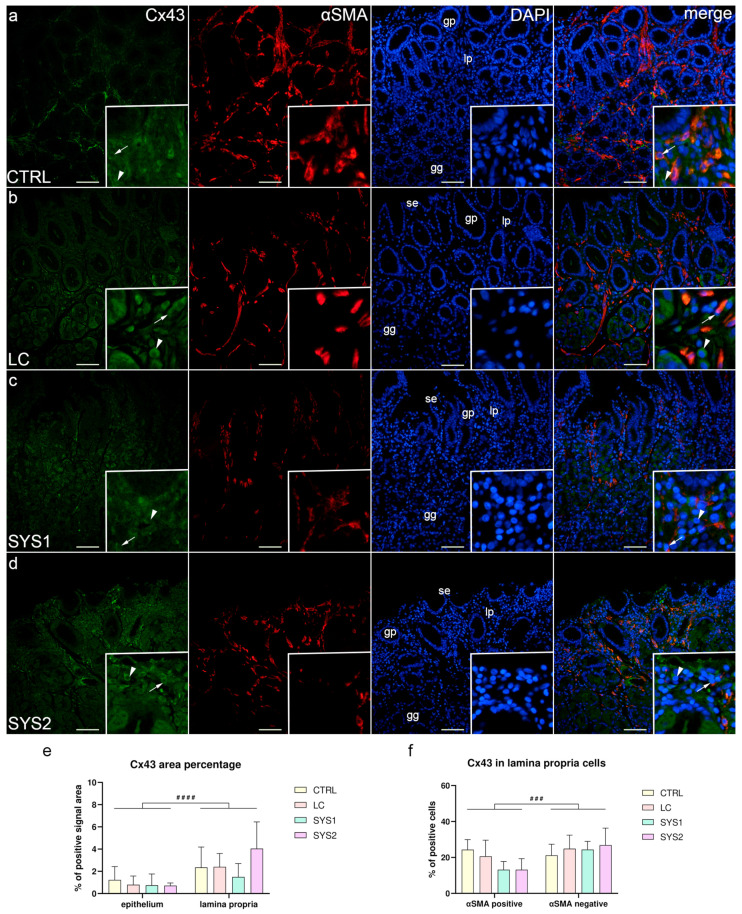
Co-expression of Cx43 and αSMA in the gastric mucosa of patients with SSc. CTRL—control samples from healthy volunteers (**a**); LC—samples from patients with limited cutaneous SSc (**b**); SYS1—samples from patients with moderate diffuse cutaneous SSc (**c**); SYS2—samples from patients with severe diffuse cutaneous SSc (**d**). se, surface epithelium; gp, gastric pits; gg, gastric glands; lp, lamina propria. Insets display Cx43 expression in lamina propria cells (**a**–**d**). Arrows mark αSMA-positive cells expressing Cx43, while arrowheads demonstrate αSMA-negative cells with Cx43 expression (**a**–**d**). DAPI staining shows all cell nuclei (**a**–**d**). Double immunofluorescence staining for Cx43 and αSMA, 200× magnification, scale bars: 200 µm. The graphs display differences in Cx43 area percentage of epithelium and lamina propria (**e**), as well as differences between αSMA-positive and -negative cells of the lamina propria (**f**). Error bars mark the standard deviation. Hashes (#) represent differences between analyzed regions/cells. ^###^ *p* < 0.001, ^####^ *p* < 0.0001.

In all sample groups, almost all epithelial structures of the gastric mucosa were negative for Cx43 staining, with the exception of some cells of the deeper parts of gastric glands that demonstrated weak Cx43 staining. While most of the cells of the lamina propria of CTRL and LC samples were Cx43-negative, strong Cx43 staining was present in about one-quarter of αSMA-positive and -negative cells (Figure 3a,b). The lamina propria of SYS1 and SYS2 samples also had mostly Cx43-negative cells; however, there were about twice as many αSMA-negative than αSMA-positive cells with strong Cx43 staining (Figure 3c,d). Although there were no statistically significant differences in overall Cx43 area percentage between the analyzed sample groups, the lamina propria had a significantly higher area percentage (*p* < 0.0001) compared to the epithelium across all sample groups (Figure 3e). No significant differences of Cx43 positivity in αSMA-positive and -negative lamina propria cells were found between sample groups. When analyzing all sample groups, a significantly higher proportion (*p* = 0.0002) of αSMA-negative cells were Cx43-positive compared to αSMA-positive cells (Figure 3f).

### 3.4. Double Immunofluorescence Staining for Panx1 and αSMA

**Figure 4:**
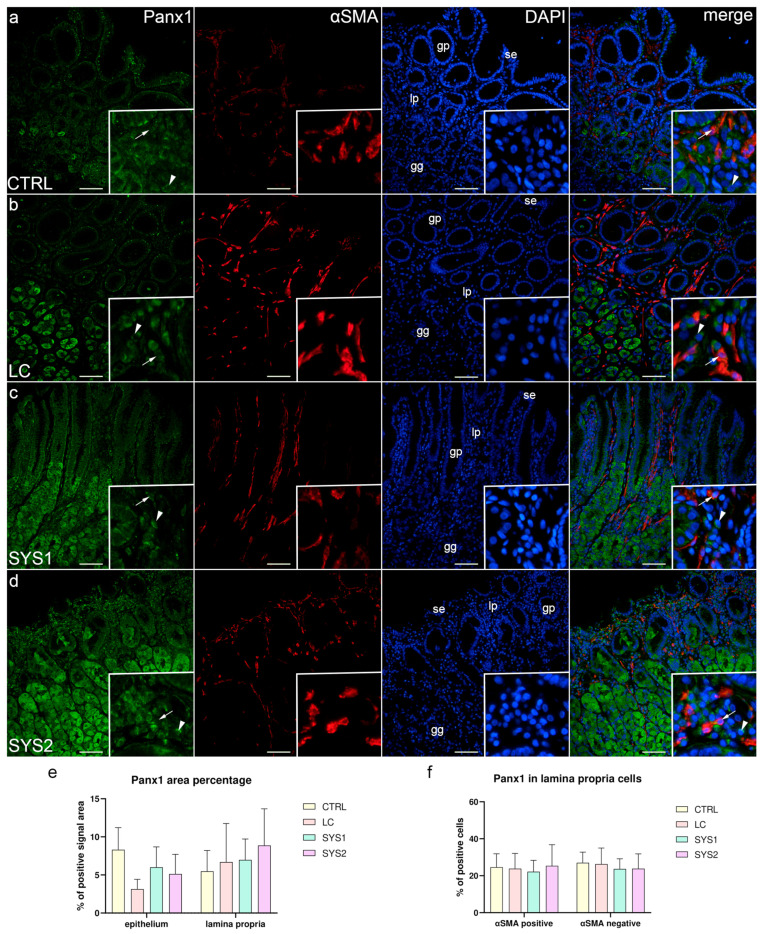
Co-expression of Panx1 and αSMA in the gastric mucosa of patients with SSc. CTRL—control samples from healthy volunteers (**a**); LC—samples from patients with limited cutaneous SSc (**b**); SYS1—samples from patients with moderate diffuse cutaneous SSc (**c**); SYS2—samples from patients with severe diffuse cutaneous SSc (**d**). se, surface epithelium; gp, gastric pits; gg, gastric glands; lp, lamina propria. Insets display Panx1 expression in lamina propria cells (**a**–**d**). Arrows mark αSMA-positive cells expressing Panx1, while arrowheads demonstrate αSMA-negative cells with Panx1 expression (**a**–**d**). DAPI staining shows all cell nuclei (**a**–**d**). Double immunofluorescence staining for Panx1 and αSMA, 200× magnification, scale bars: 200 µm. The graphs display differences in Panx1 area percentage of epithelium and lamina propria (**e**), as well as differences between αSMA-positive and -negative cells of the lamina propria (**f**). Error bars mark the standard deviation.

In all of the analyzed sample groups, strong Panx1 staining was present in the basal regions of the surface epithelium, gastric pits, and superficial parts of gastric glands. The deeper parts of gastric glands contained many cells that demonstrated diffuse Panx1 positivity. The lamina propria had several Panx1-positive cells, both αSMA-positive and αSMA-negative (Figure 4a–d). There were no statistically significant differences in area percentage between sample groups in both the epithelium and the lamina propria (Figure 4e). There were also no significant differences in the proportion of αSMA-positive and -negative cells between the analyzed sample groups (Figure 4f).

### 3.5. Comparison of Cx37, Cx40, Cx43, and Panx1 Protein Expressions

**Figure 5:**
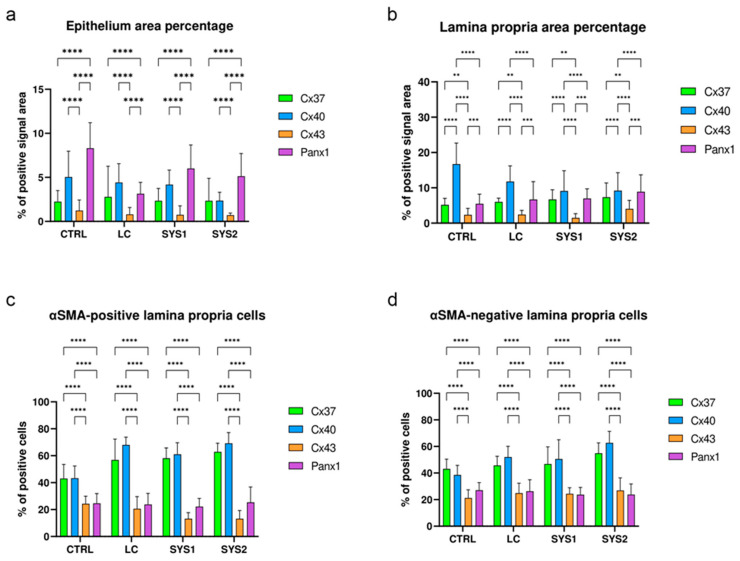
Comparison of Cx37, Cx40, Cx43, and Panx1 protein expressions in the gastric mucosa of patients with SSc. CTRL—control samples from healthy volunteers, LC—samples from patients with limited cutaneous SSc, SYS1—samples from patients with moderate diffuse cutaneous SSc, SYS2—samples from patients with severe diffuse cutaneous SSc (**a**–**d**). The graphs display differences between the analyzed proteins in area percentage of epithelium (**a**) and lamina propria (**b**), as well as in αSMA-positive (**c**) and -negative cells (**d**) of the lamina propria. Error bars mark the standard deviation. Asterisks (*) symbolize significant differences between the analyzed proteins expression. ** *p* < 0.01, *** *p* < 0.001, **** *p* < 0.0001.

We compared the area percentages and the proportions of lamina propria cells positive for the analyzed proteins across all sample groups. The area percentage of the immunofluorescence signal in epithelial structures (surface epithelium, gastric pits, and gastric glands) was the highest for Panx1, followed by Cx40, while Cx43 had the lowest area percentage. The differences were statistically significant (*p* < 0.0001) when comparing Cx43 with Panx1 and Cx40 (Figure 5a). When observing the lamina propria area percentage, Cx40 was the highest (*p* < 0.0001) compared to all three other analyzed proteins. Cx43 had the lowest area percentage, being significantly lower than both Cx37 (*p* = 0.0045) and Panx1 (*p* = 0.0002) area percentages (Figure 5b). When analyzing the proportions of positive lamina propria cells, there was a significantly higher proportion (*p* < 0.0001) of Cx37- and Cx40-positive cells compared to Cx43- and Panx1-positive cells. This was true for both αSMA-positive and -negative lamina propria cells (Figure 5c,d).

## 4. Discussion

While many advances in understanding the pathogenesis of fibrosis in SSc have been made, the interaction and involvement of different pathways leading to its appearance are still not fully determined. Multiple studies have demonstrated morphological changes consistent with fibrosis, such as collagen deposition, myofibroblast accumulation, and immune cell infiltration in the gastric mucosa of SSc patients [27,29,30]. Increased proliferation and apoptosis of both epithelial and lamina propria cells have also been described [27], as well as changes in fibrosis-associated signaling pathways in other parts of the gastrointestinal tract [31,32]. The morphological characteristics and histological differences of our samples, which were described in our previous study [27], are in line with the changes reported in other studies [29,30]. Some previous immunohistochemical studies reported only the presence of Cx43 in human gastric tissues [33,34] but not any of the other three analyzed proteins. In contrast, the tissue expression of Cx37, Cx40, and Panx1 in our healthy control samples was concordant with that described in The Human Protein Atlas (data available at v15.proteinatlas.org) [35].

In our study, we discovered alterations in Cx37 and Cx40 protein expressions in the gastric mucosa of SSc patients compared to healthy individuals. It was previously described that loss of Cx40 contributes to fibrotic changes in lung tissue [36], and we found that the protein levels (measured by area percentage) of Cx40 in the lamina propria diminished with increasing severity of SSc. Interestingly, the number of Cx40-positive cells in the lamina propria increased in SSc samples compared to healthy controls. This could be attributed to the increased proliferation of lamina propria cells, as described in a previous study [27]. Therefore, even though the number of cells expressing Cx40 was greater, the total amount (area percentage) of Cx40 in the tissue decreased in SSc samples. The increase in Cx37 protein levels was less pronounced in SSc and was not statistically significant. There was, however, a significant increase in the number of Cx37-positive lamina propria cells in the samples from patients with severe forms of dcSSc compared to healthy volunteers. However, there have not been any studies linking Cx37 to fibrosis in any tissue or organ [37].

We did not find differences in Cx43 and Panx1 protein expression in the gastric mucosa between SSc patients and healthy volunteers. The role of Cx43 in tissue fibrosis has been widely studied in different tissues (skin, liver, heart, lung, and kidney) and models, leading to differing results [37]. A study by Lu et al. demonstrated decreased Cx43 expression in fibroblasts isolated from keloid and hypertrophic scars compared to those from normal skin [38], while Asazuma-Nakamura et al. showed that Cx43 levels increased in parallel with αSMA levels and myofibroblast differentiation [39]. In our study, the absence of differences in Cx43 expression between the analyzed groups could be explained by the fact that Cx43 had a significantly weaker expression in the gastric mucosa in general compared to the other analyzed proteins. Adenosine triphosphate (ATP) release mediated by Panx1 was shown to play a role in the early events of cardiac fibrosis [40] and increased membrane permeability of apoptotic cells [41,42]. While we did not generally find statistically significant differences in Panx1 protein expression between the analyzed groups, Panx1 levels in lamina propria cells increased with the severity of SSc, which might correspond to increased fibrosis and apoptosis of gastric mucosa cells [27].

There is only one previous study that analyzed the association between Cxs and fibrosis related to SSc [43]. In their study, Stellato et al. analyzed the myocardium of a Fosl-2-overexpressing mouse model of SSc and found disorganized distribution of both Cx40 and Cx43 in the myocardium of the SSc model compared to wild-type mice. Interestingly, while the expression of Cx40 decreased, there was no significant difference in the total Cx43 expression between the SSc model and wild-type mice, which is similar to the results of our study. Considering the fact that Cx40 and Cx43 were strongly expressed in the muscle layers of the stomach [44,45], which are also the most severely affected regions of the gastric wall in SSc [46], further studies on full-thickness biopsies of the stomach could elucidate whether these proteins are altered in SSc in a comparable manner to the changes found in the myocardium.

The main limitations of our study were the small sample size and the fact that only one technique was used to estimate differences in protein levels. This was due to the samples used in this study being taken from an archive of formalin-fixed paraffin-embedded tissue as the prevalence of SSc in our region was low. Therefore, other techniques of quantifying protein expression, such as Western blot or flow cytometry, were not appropriate to use on the analyzed samples. Further studies using a greater amount of freshly gathered tissue and the aforementioned techniques are needed to confirm the results of our study.

## 5. Conclusions

There Was a discrete increase in Cx37 and a significant decrease in Cx40 protein levels in the gastric mucosa of patients with SSc compared to healthy controls. These changes were proportional to the severity of SSc, with the most prominent changes found in patients with severe dcSSc. While no differences in Cx43 and Panx1 were found in the gastric mucosa of SSc patients, studies analyzing the entire gastric wall are necessary to determine whether they play a role in SSc-associated fibrosis.

## Figures and Tables

**Table 1:** Demographic and clinical characteristics of the SSc patients and volunteers.

Characteristic	SSc Patients (*n* = 15)	Healthy Volunteers (*n* = 5)
Age (years) ^a^	54.3 ± 13.6	54.9 ± 11.2
Female sex	15 (100%)	5 (100%)
Caucasian race	15 (100%)	5 (100%)
Disease duration ^b^	13 (1–35)	N/A
Antinuclear antibody positivity	13 (86.7%)	0 (0%)
Anti-centromere antibody positivity	4 (26.7%)	0 (0%)
Anti-topoisomerase I (Scl 70) antibody positivity	9 (60%)	0 (0%)
Modified Rodnan skin score ^b^	15 (4–26)	0 (0–0)
Diffusing capacity for carbon monoxide (DLCO) ^a^	58.4 ± 22.2	N/A
Presence of interstitial lung disease	8 (53.3%)	0 (0%)
Presence of gastrointestinal tract lesions	10 (66.7%)	0 (0%)
Presence of heart involvement	3 (20%)	0 (0%)
Presence of peripheral vascular involvement	12 (80%)	0 (0%)
Presence of digital ulcers	3 (20%)	0 (0%)
Presence of skeletal muscle involvement	10 (66%)	0 (0%)
Presence of joint and tendon involvement	12 (80%)	0 (0%)

^a^ Values are expressed as the mean ± standard deviation; ^b^ values are expressed as the median (minimum–maximum); N/A—not applicable or not assessed.

**Table 2:** Primary and secondary antibodies used in the study.

	Antibodies	Host	Code No.	Dilution	Source
Primary	Anti-Cx37/GJA4	Rabbit	ab181701	1:300	Abcam (Cambridge, UK)
	Anti-Cx40/GJA5	Rabbit	ab213688	1:50	Abcam (Cambridge, UK)
	Anti-Cx43/GJA1	Goat	ab87645	1:100	Abcam (Cambridge, UK)
	Anti-pannexin 1	Rabbit	ABN242	1:300	Merck KGaA (Darmstadt, Germany)
	Anit-Smooth Muscle Actin	Mouse	M0851	1:300	Dako (Glostrup, Denmark)
Secondary	Alexa Fluor^®^ 488 AffiniPure Anti-Goat IgG (H + L)	Donkey	705-545-003	1:400	Jackson Immuno Research Laboratories, Inc., (Baltimore, PA, USA)
	Alexa Fluor^®^ 488 AffiniPure Anti-Rabbit IgG (H + L)	Donkey	711-545-152	1:400	Jackson Immuno Research Laboratories, Inc., (Baltimore, PA, USA)
	Rhodamine Red™-X (RRX) AffiniPure Anti-Mouse IgG (H + L)	Donkey	715-295-151	1:400	Jackson Immuno Research Laboratories, Inc., (Baltimore, PA, USA)

## Data Availability

The data presented in this study are available from the corresponding author upon reasonable request.
